# Brain amyloid and vascular risk are related to distinct white matter hyperintensity patterns

**DOI:** 10.1177/0271678X20957604

**Published:** 2020-09-21

**Authors:** Lene Pålhaugen, Carole H Sudre, Sandra Tecelao, Arne Nakling, Ina S Almdahl, Lisa F Kalheim, M Jorge Cardoso, Stein H Johnsen, Arvid Rongve, Dag Aarsland, Atle Bjørnerud, Per Selnes, Tormod Fladby

**Affiliations:** 1 Department of Neurology, Akershus University Hospital, Lørenskog, Norway; 2 Institute of Clinical Medicine, University of Oslo, Oslo, Norway; 3 School of Biomedical Engineering and Imaging Sciences, King’s College London, London, UK; 4 Dementia Research Centre, Institute of Neurology, University College London, London, UK; 5 Department of Medical Physics, University College London, London, UK; 6 Betanien Hospital, Bergen, Norway; 7 Department of Geriatric Psychiatry, Oslo University Hospital, Oslo, Norway; 8 Department of Neurology, University Hospital of North Norway, Tromsø, Norway; 9 Department of Clinical Medicine, Brain and Circulation Research Group, UiT The Arctic University of Norway, Tromsø, Norway; 10 Department of Research and Innovation, Haugesund Hospital, Haugesund, Norway; 11 Department of Clinical Medicine (K1), University of Bergen, Bergen, Norway; 12 Department of Old Age Psychiatry, Institute of Psychiatry, Psychology and Neuroscience, King's College London, London, UK; 13 Center for Age-Related Diseases, Stavanger University Hospital, Stavanger, Norway; 14 Department of Diagnostic Physics, Oslo University Hospital, Oslo, Norway; 15 Department of Physics, University of Oslo, Oslo, Norway

**Keywords:** Alzheimer’s, cerebrospinal fluid, cognitive impairment/decline, small vessel disease, white matter disease

## Abstract

White matter hyperintensities (WMHs) are associated with vascular risk and Alzheimer’s disease. In this study, we examined relations between WMH load and distribution, amyloid pathology and vascular risk in 339 controls and cases with either subjective (SCD) or mild cognitive impairment (MCI). Regional deep (DWMH) and periventricular (PWMH) WMH loads were determined using an automated algorithm. We stratified on Aβ1-42 pathology (Aβ+/−) and analyzed group differences, as well as associations with Framingham Risk Score for cardiovascular disease (FRS-CVD) and age. Occipital PWMH (*p* = 0.001) and occipital DWMH (*p* = 0.003) loads were increased in SCD-Aβ+ compared with Aβ− controls. In MCI-Aβ+ compared with Aβ− controls, there were differences in global WMH (*p* = 0.003), as well as occipital DWMH (*p* = 0.001) and temporal DWMH (*p* = 0.002) loads. FRS-CVD was associated with frontal PWMHs (*p* = 0.003) and frontal DWMHs (*p* = 0.005), after adjusting for age. There were associations between global and all regional WMH loads and age. In summary, posterior WMH loads were increased in SCD-Aβ+ and MCI-Aβ+ cases, whereas frontal WMHs were associated with vascular risk. The differences in WMH topography support the use of regional WMH load as an early-stage marker of etiology.

## Introduction

White matter hyperintensities (WMHs) visible on T2-weighted magnetic resonance imaging (MRI) scans are neuroimaging hallmarks of small vessel disease (SVD),^1^ but WMHs are also associated with Alzheimer’s disease (AD) dementia,^2^,^3^ as well as preclinical amyloid pathology.^4^,^5^ However, a widely accepted model for sequential AD biomarkers does not include WMHs.^6^

The amyloid hypothesis for AD proposes that amyloid precursor protein dysmetabolism and amyloid plaques lead to neurofibrillary pathology,^7^ but vascular amyloid deposits are common and were initially suggested to have a mediating role.^7^,^8^ AD and cerebrovascular disease (CVD) share risk factors,^9^ and although a definite pathomechanistic interaction is not identified, pertinent findings strongly support a vascular component in AD.^8^,^10–12^

Neuropathological studies have revealed that WMHs are of heterogeneous origin, including amyloid angiopathy, arteriolosclerosis, activated glia and axonal rarefaction,^13–15^ and thus associated with both amyloid pathology and ischemia. Frontal WMHs were recently related to age and vascular risk.^16^,^17^ Conversely, in sporadic AD dementia, there was a posterior predilection for WMHs,^18^,^19^ and parietal WMHs predicted time to dementia in a large longitudinal study.^20^ Furthermore, in asymptomatic autosomal dominant AD mutation carriers, occipital WMH volume increased more than 20 years before estimated time of symptom-onset,^21^ coinciding with altered levels of amyloid beta 1-42 (Aβ1-42) and tau in cerebrospinal fluid (CSF).

This suggests that increased posterior WMHs may be linked to AD pathology. We therefore examined whether increased WMH load could be detected also in preclinical sporadic AD cases and assessed the utility of posterior WMHs as an early-stage AD marker. Secondarily, we assessed whether frontal WMHs are more closely related to age and vascular risk factors, and whether the overall distribution supports an emerging pattern of associations between regional WMHs and underlying pathology.

## Methods

### Study population

Subjects were cases or controls enrolled in the Dementia Disease Initiation (DDI) longitudinal multicenter study in Norway in the period from December 2013 until September 2018. The criteria for inclusion were age between 40 and 80 years at baseline and a native language of Norwegian, Swedish or Danish. Exclusion criteria were brain trauma or disorder, including clinical stroke, dementia, severe psychiatric disease, severe somatic disease that might influence the cognitive functions, intellectual disability or other developmental disorders.^22^ Cases had symptoms of cognitive impairment reported by themselves or an informant and were recruited mainly by advertisement (58%), from memory clinics (24%) or from a previous study (7%). Controls were recruited from advertisement (60%) or were patients admitted to hospital for orthopedic surgery (29%). In the advertisements, individuals with first degree relatives with dementia were particularly encouraged to participate in the study. The core study protocol consisted of clinical and neuropsychological assessment, MRI and lumbar puncture, but individuals with incomplete assessments were not excluded. A subgroup consisting of controls with first degree relative with dementia and cases underwent [^18^F]flutemetamol PET in addition.

Cases with normal performance on standardized tests were classified as SCD, as defined in the framework by the working group of SCD.^23^ The NIA-AA criteria for MCI were used for cases with lower performance than expected in one or more cognitive domains, but yet preserved independence in functional ability and not fulfilling the criteria of dementia, as defined in NIA-AA guidelines.^24^,^25^ The cutoff values for SCD versus MCI were results less than 1.5 standard deviation below normative mean on either CERAD word list (delayed recall), VOSP silhouettes, TMT-B or COWAT,^22^ and the same criteria were used to classify participants with no self-reported symptoms of cognitive decline as cognitively normal (NC) or abnormal controls.

All subjects gave their written consent, and the Regional Committee for Medical and Health Research Ethics South-East evaluated (based on the Norwegian Health and Research Act and the Helsinki Declaration of 1964; revised 2013) and approved the study. All further study conduct was in line with these guidelines.

### MRI assessment and image analysis

MRI images were obtained on eight different scanners on five centers, but two of the scanners had only been used for one and three of the subjects in the study, respectively, and they were excluded. The acquisition protocol and frequency repartition on the six remaining scanners are detailed in Supplementary Table S1.

**Figure 1.**
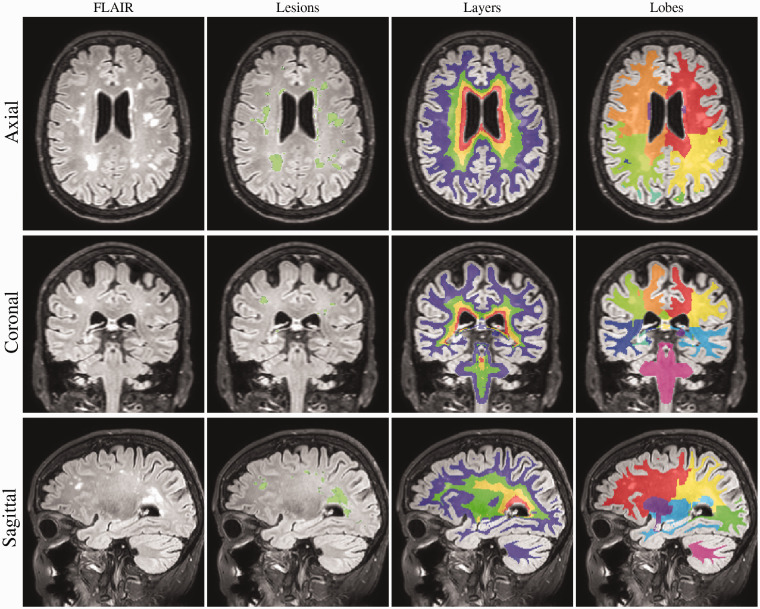
WMH segmentation. Example of the brain segmentation for one of the SCD-Aβ+ cases, a 72-year-old woman. The segmentation of WMHs is coloured green in the 2nd column. In the 3rd and 4th columns, the layers and lobes are shown, respectively. The inner and outer two layers were added to estimate periventricular and deep WMHs, respectively.

WMHs were segmented using an automatic algorithm presented elsewhere.^26^ In short, using rigidly co-registered FLAIR and T1 sequences, a Gaussian mixture model with dynamically evolving number of components is fit to the data, modelling simultaneously healthy and non-expected observations such as pathology. Anatomical information is introduced to the model through subject-specific statistical atlases obtained from a label-fusion automated framework (Geodesic Information Flows GIF).^26^ After convergence, the model is used to select candidate lesion voxels whose aggregation in connected components is automatically classified as lesion or artifacts. All segmentations were visually inspected. In order to characterize the lesion location, as described previously,^27^ the white matter was further separated in four equidistant layers between the ventricular surface and the cortical GM/WM interface, while the cortical lobar separation obtained from the label-fusion parcellation was propagated onto the WM volume to distinguish lobar sectors. The basal ganglia and thalamic regions were considered separately. See Figure 1 for the illustration of volumetric division.

### CSF analysis

Lumbar puncture was performed and CSF handled as described.^22^ CSF Aβ1-42, total tau and phosphorylated tau were determined using ELISA (Innotest β-Amyloid (1-42), Innotest hTau Ag and Innotest Phospho-Tau (181 P), Fujirebio, Ghent Belgium).

### Amyloid PET

[^18^F]flutemetamol PET images were obtained from a GE Discovery 690 PET/CT scanner. A bolus injection of 185 MBq (5 mCi) was followed by rest before positioning the subject head-first supine in the scanner. Prior to PET acquisition, a low-dose CT scan for attenuation correction was acquired. PET scanning in 3D-mode started 90 min after injection of [^18^F]flutemetamol. PET data were acquired for 20 min (four frames of five minutes). The PET detector was cylindrical, 700 mm in the xy-plane and 153 mm in the z-plane, and there were 47 slices. The matrix was 192 × 192. Slice thickness was thus 3.27 mm, original (xy plane) pixel size 3.64 mm × 3.64 mm.

### APOE genotyping

*APOE* genotyping was performed on EDTA blood samples at Akershus University Hospital (Gene Technology Division, Department of Interdisciplinary Laboratory Medicine and Medical Biochemistry) according to the laboratory’s routine protocol using real-time PCR combined with a TaqMan assay (Applied Biosystems, Thermo Fisher Scientific, Waltham, USA).

### Data analysis

In a previous study comparing CSF Aβ1-42 levels with [^18^F]flutemetamol uptake, a cutoff of 708 pg/ml classified subjects as amyloid positive (Aβ+) or negative (Aβ−) with sensitivity and specificity of 93%,^28^ and we used this cutoff in this study. For three individuals without CSF samples, Aβ status was determined by clinical evaluation of [^18^F]flutemetamol PET images. Based on this, the NC, SCD and MCI groups were further divided in Aβ+ and Aβ− groups (NC-Aβ−, NC-Aβ+, SCD-Aβ−, SCD-Aβ+, MCI-Aβ− and MCI-Aβ+). Abnormal controls and NC-Aβ+ were not included in group comparisons, due to small sample sizes.

The simple Framingham Risk Score for cardiovascular disease (FRS-CVD) was calculated for each subject, based on information about age, systolic blood pressure (SBP), use of antihypertensive medication, body mass index (BMI) and history of type 2 diabetes mellitus (DM). Because age has a large contribution to the score, it was calculated with and without the age component (FRS-CVD_woa_), to be used as parameters of vascular risk.^29^

Demographic information for continuous variables with normal distribution (age, SBP, FRS-CVD, FRS-CVD_woa_ and CSF Aβ1-42) was described by mean and standard deviation, and group differences were assessed with independent samples *t*-tests, comparing NC-Aβ− with SCD-Aβ−, SCD-Aβ+, MCI-Aβ− and MCI-Aβ+. Similarly, continuous variables with non-normal distribution (MMSE, Geriatric Depression Scale, BMI, CSF total tau and CSF phosphorylated tau) were described by median and interquartile range, and groups were compared with Mann–Whitney U tests. Categorical variables (sex, hypertension treatment, current smoking, *APOE*-ε4 status and DM) were described by frequencies and percentages and compared with Pearson’s Chi square tests across groups. *APOE*-ε4 status was defined as positive with either one or two ε4 alleles. DM was defined as positive if either diagnosis or anti-diabetic medication was confirmed in medical history or HbA1c was measured >6.5%.

Global WMH load was calculated as the sum of WMH volumes in frontal, parietal, occipital and temporal lobes, normalized against the sum of the lobar brain volumes. Regional periventricular (PWMH) and deep white matter WMH (DWMH) loads in the frontal, parietal, occipital and temporal lobes were computed by adding the inner two or the outer two layers, respectively, in these lobes, and then normalized by dividing with the corresponding regional white matter volumes. Due to right-skewed distributions, global and regional WMH loads were log-transformed. To avoid log transformation of zero values, we added 1 to the global and all regional WMH volumes.

We compared the global and regional WMH loads between Aβ− and Aβ+ stage groups by linear mixed model regression. Global or regional WMH loads were dependent variables, and age and group dummy variables were fixed independent variables, thereby comparing NC-Aβ− with SCD-Aβ−, SCD-Aβ+, MCI-Aβ− and MCI-Aβ+, adjusting for age.

We also checked for group differences in global and regional brain volumes, by using a linear mixed model with the brain volumes as dependent variables and group dummy variables and age as fixed independent variables, to assess whether possible differences due to brain atrophy contributed to the results.

To assess the associations with age and vascular risk, we performed linear mixed model regression with global or regional WMH loads as dependent variables with age and FRS-CVD_woa_ as fixed independent variables, both separately in univariable models and together in a multivariable model.

There were six scanners at five clinical centers, and we treated scanners as random effect with random intercept in all models to account for scanner and center effects. Sex was added as a covariate in the analyses of global WMH load, but having non-significant coefficients it was omitted as covariate in all analyses.

We inspected the residuals to check the validity of the regression analysis.

Because the dependent variables were correlated, we used spectral decomposition to estimate the effective number of tests,^30^ in order to correct for multiple testing. We used R version 3.6.2 (R core team 2019, Vienna, Austria) and the package “poolr” for this estimation.^31^ Stata version 15 (College Station, Texas, USA) was used in all other statistical analysis.

### Data availability

Data from this study are available upon request.

## Results

### Participant characteristics

**Figure 2.**
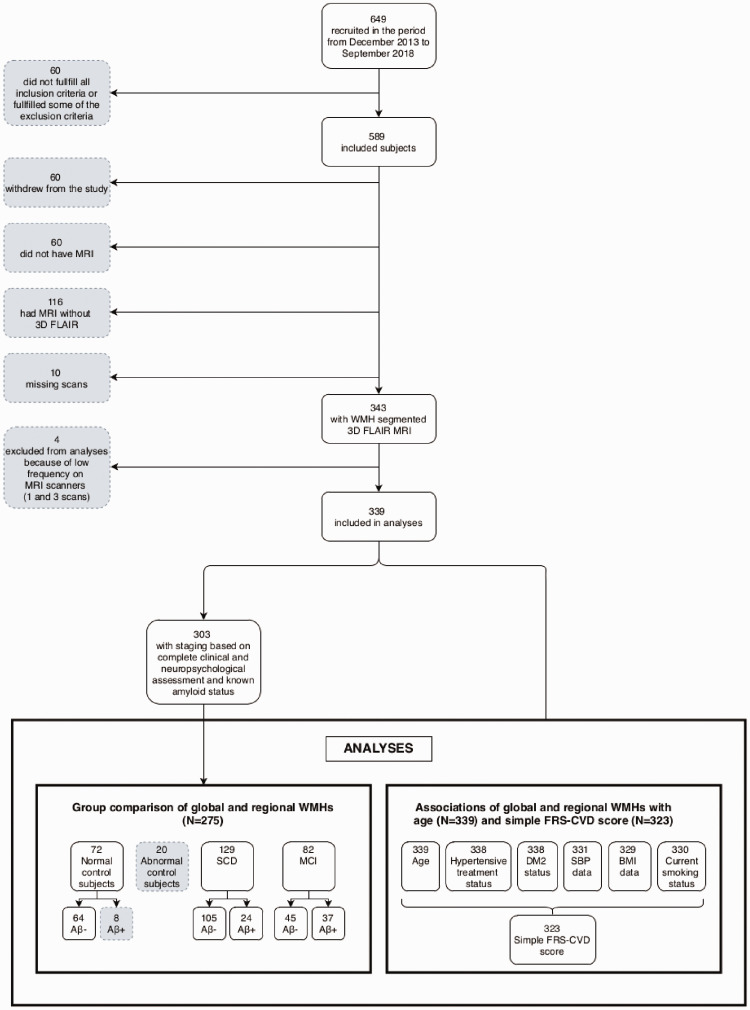
Flow chart of subject selection.

Among the 649 individuals recruited between December 2013 and September 2018, 589 fulfilled the inclusion criteria and none of the exclusion criteria. 3D FLAIR sequence was available for 343 of them, but four were excluded as they were scanned on two rarely used scanners, making them unsuitable for scanner correction. Clinical data were complete to compute the FRS-CVD score for 323 of these subjects, and amyloid status along with complete clinical and neuropsychological assessment, required for the group classification, was further known for 303 of them. A flowchart of the data selection is presented in Figure 2.

Demographics are presented in Table 1. The SCD-Aβ+, MCI-Aβ− and MCI-Aβ+ groups were significantly older than the NC-Aβ− group. All symptom groups (SCD-Aβ−, SCD-Aβ+, MCI-Aβ− and MCI-Aβ+) had higher Geriatric Depression Scale score than the NC-Aβ− group. Compared to NC-Aβ−, there were more *APOE* ε4 carriers in the SCD-Aβ+ and MCI-Aβ+ groups, and these groups had lower levels of CSF Aβ1-42 and higher levels of CSF total tau and phosphorylated tau. Both MCI-Aβ− and MCI-Aβ+ had higher FRS-CVD score than NC-Aβ-, but only MCI-Aβ− had higher FRS-CVD_woa_. As expected, both MCI-Aβ− and MCI-Aβ+ had lower MMSE score than NC-Aβ−.

**Table 1.** Demographic data for the total cohort and the clinical groups.

	Total N = 339	NC-Aβ− N = 64	SCD-Aβ− N = 105	SCD-Aβ+ N = 24	MCI-Aβ− N = 45	MCI-Aβ+N = 37
Age	63.7 (9.2)	61.0 (8.6)	61.8 (8.6)	68.6 (7.0)*	65.9 (10.2)*	70.1 (7.2)*
Female/total	187/33955.2%	29/6445.3%	65/105*61.9%	11/2445.8%	25/4555.6%	14/3737.8%
MMSE	29.0 (2.0)	29.0 (1.5)	30.0 (1.0)	29.5 (1.5)	29.0 (1.0)*	27.0 (3.0)*
*APOE* ε4 carriers	151/33844.7%	21/6333.3%	42/10540.0%	16/24*66.7%	12/4526.7%	27/37*73.0%
Geriatric depression scale	1.0 (3.0) (N = 327)	0.0 (1.0) (N = 62)	2.0 (3.0)* (N = 101)	2.0 (3.0)* (N = 24)	3.0 (4.0)* (N = 44)	2.0 (3.0)* (N = 37)
Systolic blood pressure	140.9 (18.7) (N = 331)	139.4 (16.8) (N = 64)	137.7 (16.2) (N = 101)	141.3 (17.5) (N = 24)	145.5 (19.7) (N = 45)	148.6 (21.6)* (N = 37)
Hypertension treatment	99/33829.3%	17/6426.6%	29/10527.6%	7/2429.2%	19/4542.2%	11/3729.7%
History of diabetes mellitus II	22/3386.5%	2/643.1%	8/1057.6%	0/240.0%	3/456.7%	4/3710.8%
Body mass index	25.2 (6.0) (N = 329)	26.0 (6.7) (N = 63)	24.1 (5.7)* (N = 100)	24.9 (4.7) (N = 24)	26.1 (7.1) (N = 45)	24.5 (4.5)* (N = 37)
Current smoking	48/33014.5%	6/649.4%	15/10314.6%	1/244.2%	13/45*28.9%	4/37 10.8%
FRS-CVD	15.0 (5.0) (N = 323)	13.9 (4.4) (N = 63)	14.0 (4.9) (N = 97)	15.8 (3.8) (N = 24)	17.1 (5.2)* (N = 45)	17.3 (3.9)* (N = 37)
FRS-CVD_woa_	3.6 (3.4) (N = 323)	3.1 (2.8) (N = 63)	3.2 (3.4) (N = 97)	2.8 (2.6) (N = 24)	5.1 (3.6)* (N = 45)	4.1 (3.1) (N = 37)
CSF Aβ1-42	969.1 (292.7) (N = 302)	1101.8 (208.8) (N = 64)	1099.7 (200.2) (N = 103)	556.8 (105.1)* (N = 23)	1108.9 (211.9) (N = 45)	556.5 (97.1)* (N = 37)
CSF total tau	315.0 (185.0) (N = 302)	287.5 (168.0) (N = 64)	280.0 (151.0)(N = 103)	425.0 (192.0)* (N = 23)	320.0 (231.0) (N = 45)	440.0 (532.0)* (N = 37)
CSF phosphorylated tau	52.0 (26.0) (N = 302)	48.0 (22.0) (N = 64)	49.0 (19.0) (N = 103)	67.0 (34.0)* (N = 23)	52.0 (23.0) (N = 45)	66.0 (63.0)* (N = 37)

Note: Demographic information for continuous variables with normal distribution (age, systolic blood pressure, FRS-CVD, FRS-CVD_woa_ and CSF Aβ1-42) was described by mean and standard deviation, and group differences were assessed with independent samples *t*-tests. Continuous variables with non-normal distribution (MMSE, Geriatric depression scale, body mass index, CSF total tau and CSF phosphorylated tau) were described by median and interquartile range, and group differences were assessed with Mann–Whitney U tests. Categorical variables (age, sex, hypertension treatment, current smoking, *APOE*-ε4 status and diabetes mellitus II) were described by frequencies and percentages, and group differences were assessed with chi square tests. SCD-Aβ−, SCD-Aβ+, MCI-Aβ− and MCI-Aβ+ were compared with NC-Aβ−. *p < 0.05 compared to NC-Aβ-

NC-Aβ-: amyloid negative cognitively normal control; SCD-Aβ-: amyloid negative subjective cognitive decline; SCD-Aβ+: amyloid positive subjective cognitive decline; MCI-Aβ-: amyloid negative mild cognitive impairment; MCI-Aβ+: amyloid positive mild cognitive impairment; MMSE: Mini-Mental State Examination; FRS-CVD: The simple Framingham Risk Score for cardiovascular disease; FRS-CVD_woa_: The simple Framingham Risk Score for cardiovascular disease without the age component.

**Table 2.** Comparison of global and regional WMH loads across clinical groups.

Group comparison		Difference	95% C.I.	*p* value
Global WMHs				
NC-Aβ−	SCD-Aβ−	−0.096	(−0.358, 0.166)	0.474
NC-Aβ−	SCD-Aβ+	0.509	(0.110, 0.907)	**0.012**
NC-Aβ−	MCI-Aβ−	0.290	(−0.034, 0.614)	0.079
NC-Aβ−	MCI-Aβ+	0.536	(0.182, 0.890)	**0.003**
Frontal periventricular WMHs				
NC-Aβ−	SCD-Aβ−	−0.192	(−0.493, 0.108)	0.209
NC-Aβ−	SCD-Aβ+	0.368	(−0.090, 0.825)	0.115
NC-Aβ−	MCI-Aβ−	0.255	(−0.116, 0.626)	0.178
NC-Aβ−	MCI-Aβ+	0.267	(−0.139, 0.673)	0.197
Frontal deep WMHs				
NC-Aβ−	SCD-Aβ−	−0.068	(−0.374, 0.238)	0.664
NC-Aβ−	SCD-Aβ+	0.497	(0.029, 0.964)	**0.037**
NC-Aβ−	MCI-Aβ−	0.372	(−0.007, 0.751)	0.054
NC-Aβ−	MCI-Aβ+	0.521	(0.106, 0.935)	**0.014**
Parietal periventricular WMHs				
NC-Aβ−	SCD-Aβ−	−0.213	(−0.632, 0.205)	0.318
NC-Aβ−	SCD-Aβ+	0.692	(0.057, 1.328)	**0.033**
NC-Aβ−	MCI-Aβ−	0.293	(−0.224, 0.810)	0.267
NC-Aβ−	MCI-Aβ+	0.664	(0.099, 1.229)	**0.021**
Parietal deep WMHs				
NC-Aβ−	SCD-Aβ−	−0.066	(−0.466, 0.333)	0.746
NC-Aβ−	SCD-Aβ+	0.820	(0.213,1.428)	**0.008**
NC-Aβ−	MCI-Aβ−	0.407	(−0.087, 0.901)	0.106
NC-Aβ−	MCI-Aβ+	0.765	(0.225, 1.305)	**0.005**
Occipital periventricular WMHs				
NC-Aβ−	SCD-Aβ−	0.008	(−0.231, 0.248)	0.946
NC-Aβ−	SCD-Aβ+	0.595	(0.232, 0.958)	**0.001**
NC-Aβ−	MCI-Aβ−	0.139	(−0.157, 0.435)	0.356
NC-Aβ−	MCI-Aβ+	0.415	(0.092, 0.738)	**0.012**
Occipital deep WMHs				
NC-Aβ−	SCD-Aβ−	−0.003	(−0.256, 0.250)	0.982
NC-Aβ−	SCD-Aβ+	0.577	(0.194, 0.960)	**0.003**
NC-Aβ−	MCI-Aβ−	0.091	(−0.221, 0.404)	0.567
NC-Aβ−	MCI-Aβ+	0.563	(0.222, 0.905)	**0.001**
Temporal periventricular WMHs				
NC-Aβ−	SCD-Aβ−	−0.112	(−0.437, 0.213)	0.498
NC-Aβ−	SCD-Aβ+	0.328	(−0.165, 0.821)	0.192
NC-Aβ−	MCI-Aβ−	0.225	(−0.177, 0.626)	0.272
NC-Aβ−	MCI-Aβ+	0.504	(0.066, 0.943)	**0.024**
Temporal deep WMHs				
NC-Aβ−	SCD-Aβ−	−0.051	(−0.387, 0.284)	0.764
NC-Aβ−	SCD-Aβ+	0.567	(0.056, 1.078)	**0.030**
NC-Aβ−	MCI-Aβ-	0.365	(−0.050, 0.780)	0.085
NC-Aβ−	MCI-Aβ+	0.716	(0.262, 1.170)	**0.002**

Note: We compared global and regional WMH loads between Aβ− and Aβ+ stage groups by linear mixed model regression with group dummy variables as fixed independent variables, comparing NC-Aβ− with SCD-Aβ−, SCD-Aβ+, MCI-Aβ− and MCI-Aβ+ adjusting for age. Scanner differences were treated as random effect with random intercept. Bold font denotes *p* < 0.05.

NC-Aβ−: amyloid negative cognitively normal control; SCD-Aβ−: amyloid negative subjective cognitive decline; SCD-Aβ+: amyloid positive subjective cognitive decline; MCI-Aβ−: amyloid negative mild cognitive impairment; MCI-Aβ+: amyloid positive mild cognitive impairment; WMHs: white matter hyperintensities.

### Group differences in global and regional WMH loads

**Figure 3.**
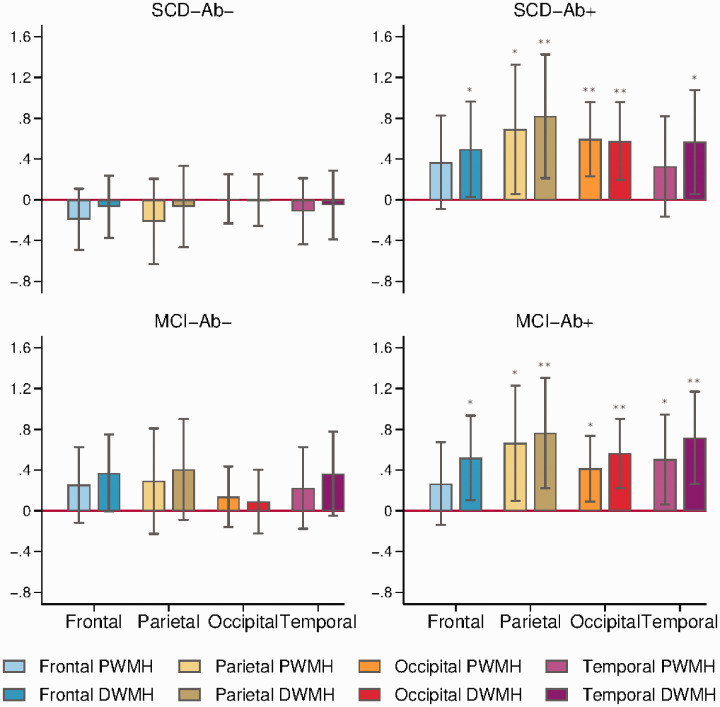
Regional WMH loads. Barplots of regression coefficients with regional WMH loads as dependent variables and group dummy variables as independent variables, showing the differences in SCD-Aβ+, MCI-Aβ− and MCI-Aβ+ compared with NC-Aβ−, adjusted for age and scanners, with error bars marking 95% confidence intervals. * *p*<0.05. ** *p*<0.01

We found that global (*p* = 0.012), occipital DWMH (*p* = 0.003), occipital PWMH (*p* = 0.001), parietal DWMH (*p* = 0.008), parietal PWMH (*p* = 0.033), temporal DWMH (*p* = 0.030) and frontal DWMH (*p* = 0.037) loads were increased in SCD-Aβ+ compared with NC-Aβ−, see Table 2 and Figure 3.

Comparing MCI-Aβ+ with NC-Aβ−, we found increased global (*p* = 0.003), occipital DWMH (*p* = 0.001), occipital PWMH (*p* = 0.012), parietal DWMH (*p* = 0.005), parietal PWMH (*p* = 0.021), temporal DWMH (*p* = 0.002), temporal PWMH (*p* = 0.024) and frontal DWMH loads (*p* = 0.014).

The difference between MCI-Aβ− and NC-Aβ− in frontal DWMHs was borderline significant (*p* = 0.054). For frontal PWMHs, there were no differences across groups.

In these analyses, there were nine dependent variables (global and regional WMH) with four group comparisons in each regression model. Because the dependent variables were correlated, we used spectral decomposition to estimate that the effective number of tests was 16 (4 × 4), yielding a multiple testing significance threshold of *p* ≤ 0.0031. The differences between SCD-Aβ+ compared with NC-Aβ− in occipital DWMH and occipital PWMH survived multiple testing correction, as did the differences between MCI-Aβ+ compared with NC-Aβ− in global WMH, occipital DWMH and temporal DWMH.

There were no differences in global or regional brain volumes between any symptom groups compared with NC-Aβ−, except for temporal periventricular brain volume in the MCI-Aβ− group (*p* = 0.031, data now shown).

### Linear regression analysis of global and regional WMHs with age and vascular risk

**Figure 4.**
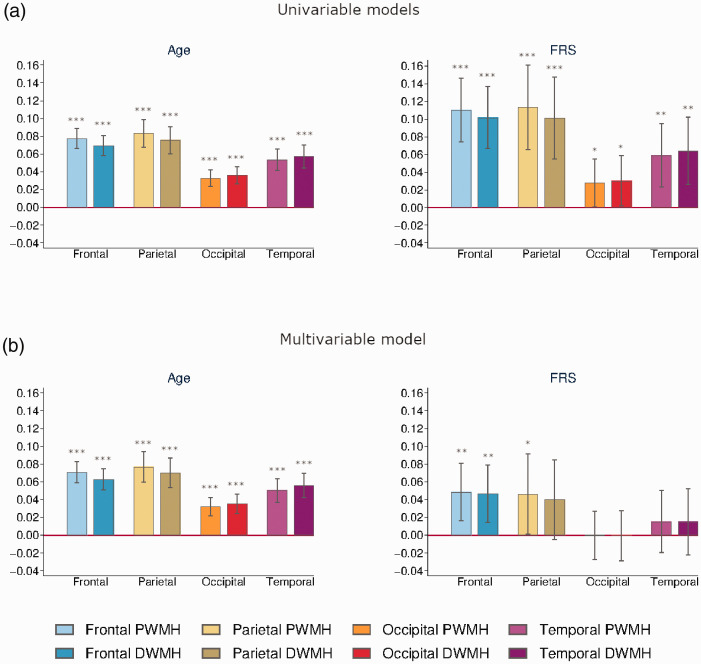
Effects of age and FRS-CVD on regional WMH. Barplots of regression coefficients with regional WMH loads as dependent variables and age and FRS-CVD_woa_ as independent variables, in univariable (a) or multivariable (b) models, all models adjusted for scanners, with error bars marking 95% confidence intervals. *p<0.05. **p<0.01. ***p<0.001.

There were significant associations between age and global and all regional WMH loads, with the highest coefficient for parietal PWMHs (β = 0.0832, *p* < 0.001) and the lowest value for occipital PWMHs (β = 0.0329, *p* < 0.001), and this did not change much in the multivariable model correcting for FRS-CVD_woa_, see Table 3 and Figure 4.

**Table 3.** Associations of global and regional WMH loads with age and FRS-CVD_woa_.

	Age			FRS-CVDwoa		
	β	95% C.I.	*p*-value	β	95% C.I.	*p*-value
Univariate models						
Global WMHs	0.0615	(0.0515, 0.0714)	**<0.001**	0.0842	(0.0532, 0.1152)	**<0.001**
Frontal PWMHs	0.0774	(0.0660, 0.0887)	**<0.001**	0.1104	(0.0743, 0.1466)	**<0.001**
Frontal DWMHs	0.0694	(0.0580, 0.0808)	**<0.001**	0.1017	(0.0666, 0.1367)	**<0.001**
Parietal PWMHs	0.0832	(0.0674, 0.0990)	**<0.001**	0.1134	(0.0656, 0.1612)	**<0.001**
Parietal DWMHs	0.0755	(0.0601, 0.0910)	**<0.001**	0.1013	(0.0550, 0.1477)	**<0.001**
Occipital PWMHs	0.0329	(0.0235, 0.0423)	**<0.001**	0.0278	(0.0006, 0.0549)	**0.045**
Occipital DWMHs	0.0361	(0.0262, 0.0459)	**<0.001**	0.0302	(0.0017, 0.0587)	**0.038**
Temporal PWMHs	0.0535	(0.0415, 0.0656)	**<0.001**	0.0591	(0.0233, 0.0948)	**0.001**
Temporal DWMHs	0.0573	(0.0444, 0.0701)	**<0.001**	0.0640	(0.0258, 0.1023)	**0.001**
Multivariable model						
Global WMHs	0.0566	(0.0460, 0.0672)	**<0.001**	0.0346	(0.0062, 0.0630)	**0.017**
Frontal PWMHs	0.0707	(0.0586, 0.0827)	**<0.001**	0.0484	(0.0162, 0.0806)	**0.003**
Frontal DWMHs	0.0629	(0.0508, 0.0749)	**<0.001**	0.0466	(0.01431, 0.0790)	**0.005**
Parietal PWMHs	0.0769	(0.0599, 0.0939)	**<0.001**	0.0462	(0.0009., 0.0915)	**0.046**
Parietal DWMHs	0.0701	(0.0534, 0.0868)	**<0.001**	0.0399	(−0.0046, 0.0845)	0.079
Occipital PWMHs	0.0321	(0.0219, 0.0424)	**<0.001**	−0.0003	(−0.0275, 0.0269)	0.984
Occipital DWMHs	0.0353	(0.0246, 0.0460)	**<0.001**	−0.0006	(−0.0290, 0.0278)	0.968
Temporal PWMHs	0.0503	(0.0372, 0.0634)	**<0.001**	0.0151	(−0.0198, 0.0500)	0.396
Temporal DWMHs	0.0559	(0.0420, 0.0698)	**<0.001**	0.0151	(−0.0220, 0.0522)	0.424

Note: We performed linear mixed model regression with global and regional WMH loads as dependent variables with age and FRS-CVD_woa_ as fixed independent variables, both separately (univariate models) and in the same model (multivariable model). Scanner differences were treated as random effect with random intercept in all models. Bold font denotes *p* < 0.05.

WMHs: white matter hyperintensities; PWMHs: periventricular white matter hyperintensities; DWMHs: deep white matter hyperintensities; FRS-CVD_woa_: The simple Framingham Risk Score for cardiovascular disease without the age component.

FRS-CVD_woa_ was significantly associated with global and all regional WMH loads, but after correcting for age, only the associations with global WMHs (β = 0.0346, *p* = 0.017) as well as frontal PWMHs (β = 0.0484, *p* = 0.003), frontal DWMHs (β = 0.0466, *p* = 0.005) and parietal PWMHs (β = 0.0462, *p* = 0.046) remained significant.

There were nine dependent variables (global and regional WMH) with two independent variables in the regression models. Bu using spectral decomposition, we estimated the effective number of tests to be 8 (4 × 2), yielding a multiple testing significance threshold of *p* ≤ 0.0063. In the multivariable model, the association with age survived multiple testing corrections in global and all regional WMHs, while the association with FRS-CVD_woa_ was only significant with frontal PWMHs and frontal DWMHs after correction for multiple testing.

## Discussion

### Main results

The main finding of this study is that occipital PWMH and DWMH burden is increased in SCD-Aβ+ compared with NC-Aβ−, also after correction for multiple testing. In MCI-Aβ+, we found increased global WMHs, occipital and temporal DWMHs compared with NC-Aβ−, after correction for multiple testing. The largest differences were seen in the deep parietal region, for both SCD-Aβ+ and MCI-Aβ+, but even though there were significant differences, they did not survive the multiple testing correction.

Only frontal PWMH and DWMH loads were associated with vascular risk assessed by FRS-CVD_woa_, after adjusting for age and correction for multiple testing.

SCD cases often seek medical help, but cognitive screening is normal, function in work and daily life may be preserved and next of kin may report no concern. Our findings suggest that increased WMH burden in SCD should raise the suspicion of AD and elicit further investigations. However, this is not in line with the current NIA-AA guidelines,^25^ stating that patients with severe WMH burden should only be diagnosed with possible (not probable) AD. None of the guidelines for preclinical or predementia AD include WMHs in their biomarker models,^24^,^32^ and extensive WMH burden is an exclusion criterion in several recent clinical trials in predementia AD.

### Supporting results

Cognitive function is associated with WMH load,^33^ and WMH volume was recently found to predict amyloid positivity in cognitively normal individuals.^4^ In the present study, we divided the cognitively normal group in asymptomatic controls and subjective cognitive decline and further stratified on evidence for amyloid pathology. To our knowledge, we are the first to describe increased occipital WMH burden in amyloid positive SCD subjects compared with asymptomatic cognitively normal amyloid negative controls.

Our findings of increased posterior WMHs in preclinical AD are consistent with previous research. In the Dominantly Inherited Alzheimer Network (DIAN) cohort of asymptomatic carriers of dominant AD genes, increased occipital WMHs occurred more than two decades before estimated time of symptom onset, coinciding with Aβ and phosphorylated tau pathology.^21^ Also, only parietal WMHs predicted time to incident AD dementia in a large cohort of non-demented elderly.^20^ On the other hand, frontal WMHs have been associated with age and cardiovascular risk, and then especially hypertension, in two recent studies.^17^,^18^

### Conflicting findings

A recent systematic review and meta-analysis found an overall association between WMHs and evidence for amyloid deposition,^4^ but there are also contradicting findings.^34^,^35^ Frontal WMHs as well as parietal WMHs were associated with amyloid positivity in a cohort of non-demented elderly.^36^ However, mean age was more than 10 years higher in this cohort, and twice as many cases had hypertension, possibly explaining more frontal WMHs. Diverging results may be explained by different ways of assessing WMHs and heterogeneity in cohorts regarding age, heredity, clinical diagnoses, stage of disease and sample size, reflected by the various selection strategies, as well as differences in categorization thresholds and covariate models. In studies with groups based on clinical diagnosis, misdiagnosis or mixed pathological conditions may obscure the analyses. Because WMH aetiology is heterogeneous, total burden likely reflects both amyloid and non-amyloid SVD, thus reducing the probability of finding statistical relationships.

### Interpretation

Chronic cerebral ischemia is associated with WMHs, but the direct mechanisms are unclear. In longitudinal studies, WMHs predate a reduction in cerebral perfusion and vice versa.^37^ Whether WMHs in AD and non-amyloid SVD represent similar or different pathogenetic mechanisms or not, remains uncertain. Several studies have underlined the heterogeneity of WMHs,^13^,^14^ that has been associated with SVD of both non-amyloid and amyloid type.^15^ We have previously shown that WMHs display reduced metabolism and more severe loss of integrity in Aβ+ compared to Aβ− cases, using glucose-PET and diffusion tensor imaging.^38^,^39^ Parietal WMHs were recently connected to Wallerian degeneration in AD, putatively secondary to cortical neurofibrillary tangles,^14^ but a posterior WMH distribution in AD could also be linked to the predilection for cerebral amyloid angiopathy (CAA) in this region.^40^

The frequent coexistence of WMHs with AD pathology has raised the question of an interaction between CVD and AD.^41^ Animal studies in mice have shown reduced clearance mechanisms of Aβ in *APOE*ε4 carriers, both via perivascular pathways and across the blood–brain barrier (BBB), increasing the Aβ depositions along vessel walls, such as in CAA.^42^,^43^ A prevailing hypothesis is that the interstitial fluid enters the perivascular route at the level of the capillaries and then follows along the vasculature in the basement membrane of arteries towards the subarachnoid space, and general aging of the vasculature and cerebrovascular disease can affect this mechanism of Aβ clearance.^43^ Thus, tortuosity, stiffness and changes in pulsations of arteries may reduce the effectiveness of this drainage pathway and cause build-up of amyloid depositions, potentially adding to the vascular pathology. Increased arterial stiffness is associated with both increased WMH load and cortical Aβ deposition, possibly mediating the effect of cardiovascular risk factors.^44^

Other possible mechanisms of interaction could be Aβ affecting components of the neurovascular unit, causing dysfunction of the BBB and dysregulation of cerebral blood flow.^45^,^46^ For instance, pericytes regulate capillary diameter,^47^ but are vulnerable to Aβ.^48^,^49^ With the capillary bed contributing the most to the cerebrovascular resistance, pericyte degeneration could be a substantial factor in cerebral hypoperfusion in AD.^49^,^50^ In addition, the common finding of arteriosclerosis and lipohyalinosis in neuropathological studies of AD, even in very early stages,^8^ along with the observation of vascular Aβ deposition in spontaneously hypertensive stroke-prone rats,^12^ suggests that non-amyloid small vessel disease could also cause Aβ build-up. As such, feed-forward mechanisms may exist, causing vicious cycles of compromised vascular health and Aβ aggregation.^51^

### Strengths and limitations

A strength of this study is the use of CSF sampling to reveal Aβ status in cognitively normal individuals (NC and SCD) as well as MCI. Newly developed volumetric measures of WMHs in cerebral regions promote more accurate analysis than visual scales that have been frequently used.

One of the limitations in this study is that we have only used CSF Aβ1-42 as a biomarker of predementia AD, corresponding to Stage 1 in the NIA-AA recommendations for preclinical AD.^32^ This might also embrace cases with CAA,^52^ and while there is considerable overlap between CAA and AD, they are regarded as distinct clinical conditions. The groups were not age-matched, and six different MRI scanners were used, but we corrected for age and scanners in all analysis to compensate for this. There is a probable selection bias in our cohort towards individuals with increased heredity for dementia and a higher proportion of *APOE* ε4 carriers compared to the general population, also among the controls, as a consequence of our recruitment strategy. One can speculate whether this has had an influence on the spatial pattern of WMHs. In some studies, topographical effects of *APOE* on WMHs have been presented, although the findings are somewhat inconsistent.^16^,^53^ Finally, the cross-sectional design limits the interpretation of the findings.

## Conclusions

We found increased occipital WMH load in SCD-Aβ+ compared with asymptomatic cognitively normal Aβ− controls. This is in accordance with findings in autosomal dominant AD mutation carriers, and supports the utility of posterior WMHs as a marker of early-stage AD. Only frontal WMH load was associated with vascular risk factors, after controlling for age, and these findings of differences in WMH topography support an emerging pattern of associations between regional WMHs and underlying pathology.

## Supplemental Material

sj-pdf-1-jcb-10.1177_0271678X20957604 - Supplemental material for Brain amyloid and vascular risk are related to distinct white matter hyperintensity patterns Click here for additional data file. Supplemental material, sj-pdf-1-jcb-10.1177_0271678X20957604 for Brain amyloid and vascular risk are related to distinct white matter hyperintensity patterns by Lene Pålhaugen, Carole H Sudre, Sandra Tecelao, Arne Nakling, Ina S Almdahl, Lisa F Kalheim, M Jorge Cardoso, Stein H Johnsen, Arvid Rongve, Dag Aarsland, Atle Bjørnerud, Per Selnes and Tormod Fladby in Journal of Cerebral Blood Flow & Metabolism
