# Supplementary material for: Brain amyloid and vascular risk are related to distinct white matter hyperintensity patterns
Source: J Cereb Blood Flow Metab. 2020 Sep 21;41(5):1162–74. doi: 10.1177/0271678X20957604 (PMC8054718; doi:10.1177/0271678X20957604)
Supplement: sj-pdf-1-jcb-10.1177_0271678X20957604 - Supplemental material for Brain amyloid and vascular risk are related to distinct white matter hyperintensity patterns [file sj-pdf-1-jcb-10.1177_0271678X20957604.pdf]

Table S1. MRI scanner system parameters and number of scans.

Scanner system	Scanner parameters		Number of patients scanned
Siemens Avanto 1.5T (Siemens Medical Solutions, Erlangen, Germany)	3D T1-weighted magnetization-prepared rapid gradient–echo sequence (TR/TE/TI/FA = 1700 ms/2.42 ms/1000 ms/15°, matrix = 256 × 256, 144 slices, thickness = 1.2 mm, in-plane resolution of 1.0 mm × 1.0 mm)	3D FLAIR sequence (TR/TE/TI = 6000 ms/363 ms/2200 ms, slice thickness = 1.2 mm, in-plane resolution of 1.2 mm × 1.2 mm)	21
Siemens Skyra 3T (Siemens Medical Solutions, Erlangen, Germany)	3D T1 magnetization-prepared rapid gradient–echo sequence (TR/TE/TI/FA = 2300 ms/2.98 ms/900 ms/9°, matrix = 256 × 256, 176 slices, thickness = 1.2 mm, in-plane resolution of 1.0 mm × 1.0 mm)	3D FLAIR sequence (TR/TE/TI= 5000 ms/394 ms/1800 ms, slice thickness = 1.2 mm, in-plane resolution of 1.0 mm × 1.0 mm)	38
Philips Ingenia 3T (Philips Medical Systems, Best, the Netherlands)	3D T1-weighted turbo field echo sequence (TR/TE/TI/FA=4.7ms/2.3ms/853ms/8°, matrix=256×256, 184 slices, thickness=2.0 mm, in-plane resolution of 1.0 mm×1.0mm)	3D FLAIR sequence (TR/TE/TI=4800 ms/320ms/1650ms, slice thickness=2.0mm, in plane resolution of 1.0 mm × 1.0 mm)	63
Philips Achieva 3T (Philips Medical Systems, Best, the Netherlands)	3D T1-weighted turbo field echo sequence (TR/TE/TI/FA = 6.7 ms/3.1 ms/853 ms/8°, matrix = 256 × 256, 170 slices, thickness = 1.2 mm, in-plane resolution of 1.0 mm × 1.0 mm)	3D FLAIR sequence (TR/TE/TI=8000 ms/418ms/2400ms, slice thickness=1.2 mm, in plane resolution of 1.0 mm × 1.0 mm)	70
Philips Ingenia 1.5T (Philips Medical Systems, Best, the Netherlands)	3D T1-weighted turbo field echo sequence (TR/TE/TI/FA = 7.55 ms/3.43 ms/930 ms/8°, matrix = 256 × 256, 157 slices, thickness = 1.0 mm, in-plane resolution of 1.0 mm × 1.0 mm)	3D FLAIR sequence (TR/TE/TI=4800ms/356ms/1660 ms, slice thickness=1.2 mm, in plane resolution of 1.0 mm × 1.0 mm)	71
GE Optima 1.5 T GE Healthcare, Chicago, IL	3D T1-weighted fast spoiled gradient–echo sequence (TR/TE/TI/FA = 11.3 ms/5.04 ms/500 ms/10°, matrix = 256 × 256, 156 slices, thickness = 1.2 mm, in-plane resolution of 1.0 mm × 1.0 mm)	3D FLAIR sequence (TR/TE/TI=6000ms/135.6 ms/1840 ms, slice thickness=1.2 mm, in plane resolution of 1.0 mm × 1.0 mm)	76
